# A Retrospective Analysis of the Clinical and Epidemiological Characteristics of COVID-19 Patients in Henan Provincial People's Hospital, Zhengzhou, China

**DOI:** 10.3389/fmed.2020.00286

**Published:** 2020-06-05

**Authors:** Jing Zhao, Hao-Yu Gao, Zi-Yi Feng, Qi-Jun Wu

**Affiliations:** ^1^Department of Laboratory, Henan Provincial People's Hospital, Peolple's Hospital of Zhengzhou University, Zhengzhou, China; ^2^Department of Student, China Medical University, Shenyang, China; ^3^Department of Clinical Epidemiology, Clinical Research Center, Shengjing Hospital of China Medical University, Shenyang, China

**Keywords:** coronavirus disease 2019, SARS-CoV-2, epidemiology, clinical characteristics, traditional Chinese medicine

## Abstract

**Objective:** This study evaluated the clinical and epidemiological characteristics of patients with confirmed coronavirus disease 2019 (COVID-19).

**Methods:** This retrospective study evaluated 29 patients with confirmed COVID-19 infection admitted to Henan Provincial People's Hospital between January 27 and February 27, 2020, with follow-up until April 01, 2020.

**Results:** The median age of the patients was 56 years. Nineteen (19/29; 65.5%) had underlying conditions including cardiovascular disease, digestive disease, or type 2 diabetes mellitus. Twenty-two (22/29; 76%) had close contact with acquaintances or family members who were confirmed or probable COVID-19 cases. Many patients had white blood cell counts with abnormal neutrophil and lymphocyte numbers, abnormal hemoglobin concentration, coagulation profiles, and blood biochemistry, and increased infection markers. Mottling and multiple ground-glass opacities were seen in X-ray images of 19 patients (19/29; 65.5%). Most patients (23/29; 79.8%) received supplemental oxygen therapy and antibiotics (23/29; 79.8%) in addition to traditional Chinese medicines (26/29; 89.7%). The most frequent presenting symptoms were fever, cough, and sputum production. One patient, an 86-years-old woman with more than one underlying disease, died during follow-up. Patients with severe disease were significantly older and more likely to have been transferred from other healthcare facilities than those with mild disease. Anemia, decreased activated partial thromboplastin time, calcium, and albumin, and increased D-dimer and interleukin-6 were more frequent in severe disease. Need of oxygen therapy, mechanical ventilation, intravascular immunoglobulin, and duration of antibiotic therapy were increased in those with severe disease.

**Conclusions:** Significant differences in demographical and clinical characteristics were observed in patients with moderate and severe COVID-19.

## Introduction

Human infections caused by a novel SARS-CoV-2 corona virus first appeared in Wuhan, China, in December 2019, and by early 2020 the outbreak of coronavirus disease 2019 (COVID-19) had progressed to a global pandemic. The initial cases of COVID-19 were described as a pneumonia of unknown etiology. The first four patients were exposed in the Huanan (Southern China) Seafood Market (1). Initially, the pneumonia presented with symptoms of respiratory infection, but some patients experienced severe disease that ultimately progressed to acute respiratory distress syndrome (ARDS), or even death. COVID-19 has had a great impact on Wuhan, other regions of China, and most other countries worldwide. As of April 01, 2020, there have been 82,631 confirmed infections in all 34 Chinese Provincial Administrative Regions that caused 3,321 deaths. The latest World Health Organization (WHO) Situation Report includes 823,626 infections and 40,598 deaths in more than 200 countries, territories, and areas (2). The pandemic is a serious threat to health worldwide. The basic reproduction number of SRAS-CoV-2 was estimated to be between 2.24 and 3.58 (3, 4).

Coronaviruses that are closely related to SARS-CoV can be found in bats, and there is strong evidence that SARS-CoV came from Chinese horseshoe bats (5). Similarly, MERS-CoV was transmitted by mainly dromedary camels, but it has been found in more than 14 bat species and may also have originated from bats (6). Evolutionary analysis indicates that bats are the most likely host of SARS-CoV-2 and that the virus was transmitted to humans by some unknown intermediate host that was sold at the Hunan Seafood Market (7). Both SARS-CoV-2 and SARS-CoV share angiotensin-converting enzyme 2 (ACE2) as a functional receptor, which mediates binding to host cells and disease transmission (8–10). Located in the north of Hubei province, Henan is one of the six provincial administrative regions bordering Hubei, and one of several most populated provinces and important integrated transportation hubs in China. The region is connected to Hubei in many ways on a considerable level (11). Huge population numbers frequently flow between the two provinces, especially from/to Wuhan, as the provincial capital and where original infections were reported. SARS-CoV-2 infections were reported in Henan shortly after the beginning of the outbreak in Hubei and has the third largest number of confirmed cases on a provincial level, with more than 1,200 infections and 22 deaths (12). Although the COVID-19 outbreak is spreading rapidly, information about infections imported to provinces outside of Hubei is limited. This study describes the epidemiological, laboratory, and clinical features of 29 confirmed COVID-19 patients admitted to Henan Provincial People's Hospital, Zhengzhou, which was one of three hospitals in Henan designated to admit patients with severe COVID-19 infections.

## Materials and Methods

### Patients and Study Design

The emergence of COVID-19 cases in Henan province alerted local health authorities, and the Henan provincial government prohibited travel and began admitting all patients with severe infections to Henan Provincial People's Hospital beginning on January 17, 2020 (13). Despite strict preventive and control measures, such as banning provincial public transportation and rules on wearing protective face masks in public, imported cases continued to emerge in Henan. The first 29 patients who were diagnosed with severe COVID-19 following the WHO interim guidelines (14) and admitted for treatment were included in this study. The admission dates were Jan 27 to Feb 27, 2020, and the cutoff for data collection was April 01, 2020.

### Data Collection

The clinical characteristics, laboratory findings, treatment regiments, chest X-rays, and computed tomography (CT) images were retrieved from electronic medical records. The records were verified by the Henan Provincial Center for Disease Control and Prevention to avoid possible bias. Epidemiological information including recent contacts and travel, time of illness onset, first admission, hospital transfers, and discharges were retrieved from medical records or interviews with attending physicians. Patients were transferred to Henan Provincial People's Hospital from other parts of Henan because of the severity of their infection, or were admitted locally in Zhengzhou. SARS-CoV-2 infection was confirmed by a positive reverse-transcription polymerase chain reaction (RT-PCR) assay. Data collection was carried out independently by two investigators and reviewed by two different investigators.

### Laboratory Confirmation and Treatment

Patient sputum for assays of viral, bacterial, or fungal infections was collected on admission. The initial evaluation included general status, age and gender, laboratory tests including complete blood count, coagulation assays, serum biochemistry (including alanine aminotransferase, aspartate aminotransferase, creatinine, total bilirubin, blood urea nitrogen, creatine kinase, lactate dehydrogenase, myoglobin, and glucose), and electrolytes. Symptoms, signs, travel, and contact history within the 14 days before onset, hospital transfer information (if any), chronic diseases, and treatment and clinical outcomes were also collected. Following the diagnosis and treatment guidelines (fifth edition) of the Chinese National Health Commission (15), patients were discharged after two independent negative RT-PCR assays with an interval of at least 24 h.

COVID-19 severity was graded following the diagnostic criteria of the National Health Commission of the People's Republic of China (fifth edition) (15). Mild cases were asymptomatic or presented with few symptoms; imaging found no evidence of pneumonia. Moderate cases presented with fever, symptoms of respiratory tract infection, and signs of pneumonia on imaging. Severe cases presented with any of the following: respiratory rate ≥30 breaths/min, peripheral oxygen saturation (SpO_2_) ≤93%, or oxygen partial pressure/fractional inspired oxygen (PaO_2_/FIO_2_) ≤300 mmHg (1 mmHg = 1.133 kPa). Critical cases presented with any of the following: respiratory failure with need of mechanical ventilation, shock, or other organ failure that required ICU admission.

### Statistical Analysis

Statistical analysis was performed with SPSS 26.0 (IBM Corp., Armonk, NY, USA). The values of continuous variables were reported as medians and interquartile range (IQR). Categorical variables were reported as numbers and percentages. The Mann–Whitney *U*-test was used to compare differences in continuous variables. Values of categorical variables were compared by chi-square or Fisher's exact tests. All *P*-values were two-sided and those that were <0.05 were considered significant.

### Ethical Approval

The study was approved by the Ethics Commission of the Henan Provincial People's Hospital (No. 20200090) and was conducted following the ethical principles of the World Medical Association Declaration of Helsinki.

## Results

### Patient Characteristics and Epidemiological Data

The baseline clinical and demographic characteristics are shown in Table 1. The median age at diagnosis was 56 (IQR, 31.5–66.0). Nineteen (19/29; 65.5%) had underlining diseases. The most frequent were cardiovascular disease (10/29; 34%), digestive system disease (8/29; 27.5%), and type 2 diabetes mellitus (7/29; 24%). Eight patients (8/29; 27.5%) had more than one underlying disease. Nine (9/29; 31%) had traveled to Wuhan or Hubei. Twenty-two (22/29; 76%) patients had close contact with acquaintances or family members who were confirmed or probable COVID-19 cases, and the majority had been transferred from another hospital. When stratified by disease severity, patients with severe disease tended to be significantly older and were more likely to have been transferred from another healthcare facility (*P* < 0.05).

**Table 1:** Baseline clinical and demographic characteristics of the COVID-19 patients.

**Characteristics**	**All patients**	**Severity**		***P*-value**
		**Moderate**	**Severe**	
**Number of cases**	29	8	21	
**Age at diagnosis, median (IQR)**	56.0 (31.5–66.0)	31.0 (25.8–53.3)	59.0 (48.5–75.0)	<0.05
**Gender, no. (%)**				0.11
Male	14 (48.3)	2 (25)	13 (61.9)	
Female	15 (51.7)	6 (75)	8 (38.1)	
**Underlining diseases, no. (%)**				0.39
Yes	19 (65.5)	4 (50)	15 (71.4)	
No	10 (34.5)	4 (50)	6 (28.6)	
**Underlining disease types, no. (%)**				N/A
Cardiovascular disease	10 (34.5)	1 (12.5)	9 (42.9)	
Type 2 diabetes mellitus	7 (24.1)	0	7 (33.3)	
Respiratory system disease	4 (13.8)	0	4 (19)	
Digestive system disease	8 (27.6)	3 (37.5)	5 (23.8)	
Parkinson's disease	1 (3.4)	0	1 (4.8)	
Autoimmune disease	1 (3.4)	0	1 (4.8)	
More than one disease	8 (27.6)	0	8 (38.1)	
**A history of traveling to Wuhan or Hubei, no. (%)**				0.68
Yes	9 (31)	3 (37.5)	6 (28.6)	
No	20 (69)	5 (62.5)	15 (71.4)	
**Close contact with a confirmed or probable case of nCoV infection, no. (%)**				1.00
Yes	22 (75.9)	6 (75)	16 (76.2)	
No	7 (24.1)	2 (25)	5 (23.8)	
**Presence in a healthcare facility where nCoV infections have been managed, no. (%)**				0.55
Yes	4 (13.8)	0	4 (19)	
No	25 (86.2)	8 (100)	17 (81)	
**Transferred patients, no. (%)**				<0.05
Yes	20 (69)	2 (25)	18 (85.7)	
No	9 (31)	6 (75)	3 (14.3)	
**Days from illness onset to first admission, median (IQR)**	3.0 (0.5–5.0)	N/A	3.0 (0.5–5.0)	N/A

*COVID-19, coronavirus disease 2019; IQR, interquartile range; nCoV, novel coronavirus; N/A, not available*.

*P-values are from Mann–Whitney U-tests, chi-square, or Fisher's exact tests that compared the moderate- and severe-disease groups*.

### Laboratory, CT, and X-Ray Evaluation

The laboratory and imaging results are shown in Table 2. Fifteen patients (15/29; 52%) had lymphopenia, six (6/29; 21%) had leukopenia, and six had leukocytosis. Sixteen (16/29; 55%) had low hemoglobin and seven (7/29; 24%) had low platelet counts. Coagulation abnormalities included increased D-dimer in 14 patients (14/29; 48%) and low prothrombin and activated partial thromboplastin time in 13 patients (13/29; 45%). Assays of infection markers found that C-reactive protein was increased in 23 patients (23/29; 79%), serum ferritin was increased in 16 (16/29; 55%), procalcitonin was increased in six (6/29; 21%), and the erythrocyte sedimentation rate was increased in seven (7/29; 24%). A few patients had electrolyte disorders, but 24 (24/29; 83%) had decreased calcium levels. Bacterial and fungal cultures were done if infections were suspected. Seven patients (7/29; 24%) were diagnosed with bacterial coinfections, including six lung and one urinary system infection. Four (4/29; 13.8%) had fungal infections. Chest CT and/or X-ray imaging showed bilateral pneumonia in 23 patients (23/29; 79%) with multiple mottling and ground-glass opacity in 19 (19/29; 65.5%). Five (5/29; 17%) had unilateral pneumonia. When stratified by severity, we found that patients with severe infections were more likely to have anemia, decreased activated partial thromboplastin time, calcium, and albumin, and increased D-dimer and interleukin-6 (*P* < 0.05).

**Table 2:** Laboratory, chest CT, and X-ray characteristics of the COVID-19 patients.

**Characteristics**	**All patients (*n* = 29)**	**Severity**		***P*-value**
		**Moderate (*n* = 8)**	**Severe (*n* = 21)**	
**LABORATORY TEST**				
**White blood cell count (×10/L; normal range 3.5–9.5)**				0.15
Increased	6 (20.7)	0	6 (28.6)	
Normal	17 (58.6)	5 (62.5)	12 (57.1)	
Decreased	6 (20.7)	3 (37.5)	3 (14.3)	
**Neutrophils (×10/L; normal range 1.8–6.3)**				0.09
Increased	6 (20.7)	0	6 (28.6)	
Normal	18 (65.1)	5 (62.5)	13 (61.9)	
Decreased	5 (17.2)	3 (37.5)	2 (9.5)	
**Lymphocytes (×10/L; normal range 1.1–3.2)**				0.43
Normal	14 (48.3)	5 (62.5)	9 (42.9)	
Decreased	15 (51.7)	3 (37.5)	12 (57.1)	
**Platelets (×10/L; normal range 125.0–350.0)**				0.66
Increased	3 (10.3)	1 (12.5)	2 (9.5)	
Normal	19 (65.5)	6 (75)	13 (61.9)	
Decreased	7 (24.1)	1 (12.5)	6 (28.6)	
**Hemoglobin (g/L; normal range 130.0–175.0)**				<0.05
Increased	0	0	0	
Normal	13 (44.8)	7 (87.5)	6 (28.6)	
Decreased	16 (55.2)	1 (12.5)	15 (71.4)	
**COAGULATION PROFILE, NO. (%)**				
**Activated partial thromboplastin time (s; normal range 28.0–43.5)**				<0.05
Increased	2 (6.9)	0	2 (9.5)	
Normal	14 (48.3)	8 (100)	6 (28.6)	
Decreased	13 (44.8)	0	13 (61.9)	
**Prothrombin time (s; normal range 11.0–17.0)**				0.80
Increased	1 (3.4)	0	1 (4.8)	
Normal	15 (51.7)	4 (50)	11 (52.4)	
Decreased	13 (44.8)	4 (50)	9 (42.9)	
**D-dimer (μg/L; normal range 0.0–0.5)**				<0.05
Increased	14 (48.3)	1 (12.5)	13 (61.9)	
Normal	15 (51.7)	7 (87.5)	8 (38.1)	
**SERUM ELECTROLYTE CONCENTRATION, NO. (%)**				
**Potassium (mmol/L; normal range 3.5–5.5)**				0.64
Increased	1 (3.4)	0	1 (4.8)	
Normal	26 (89.7)	7 (87.5)	19 (90.5)	
Decreased	2 (6.9)	1 (12.5)	1 (4.8)	
**Sodium (mmol/L; normal range 135.0–145.0)**				0.44
Increased	3 (10.3)	0	3 (14.3)	
Normal	21 (72.4)	7 (87.5)	14 (66.7)	
Decreased	5 (17.2)	1 (12.5)	4 (19)	
**Calcium (mmol/L; normal range 2.2–2.6)**				<0.05
Increased	0	0	0	
Normal	5 (17.2)	4 (50)	1 (4.8)	
Decreased	24 (82.8)	4 (50)	20 (95.2)	
**Chloride (mmol/L; normal range 95–105)**				0.24
Increased	13 (44.8)	2 (25)	11 (52.4)	
Normal	16 (55.2)	6 (75)	10 (47.6)	
**BLOOD BIOCHEMISTRY, NO. (%)**				
**Alanine aminotransferase (U/L; normal range 9.0–50.0)**				0.32
Increased	3 (10.3)	0	3 (14.3)	
Normal	24 (82.8)	8 (100)	16 (76.2)	
Decreased	2 (6.9)	0	2 (9.5)	
**Aspartate aminotransferase (U/L; normal range 15.0–40.0)**				0.17
Increased	6 (20.7)	0	6 (28.6)	
Normal	22 (75.9)	8 (100)	14 (66.7)	
Decreased	1 (3.4)	0	1 (4.8)	
**Albumin (g/L; normal range 40.0–55.0)**				<0.05
Normal	6 (20.7)	4 (50)	2 (9.5)	
Decreased	23 (79.3)	4 (50)	19 (90.5)	
**Total bilirubin (μmol/L; normal range 5.0–21.0)**				0.65
Increased	2 (6.9)	0	2 (9.5)	
Normal	23 (79.3)	7 (87.5)	16 (76.2)	
Decreased	4 (13.8)	1 (12.5)	3 (14.3)	
**Blood urea (mmol/L; normal range 2.5–7.1)**				0.12
Increased	6 (20.7)	0	6 (28.6)	
Normal	21 (72.4)	8 (100)	13 (61.9)	
Decreased	2 (6.9)	0	2 (9.5)	
**Serum creatinine (μmol/L; normal range 44.0–104.0)**				0.30
Increased	1 (3.4)	0	1 (4.8)	
Normal	19 (65.5)	7 (87.5)	12 (57.1)	
Decreased	9 (31)	1 (12.5)	8 (38.1)	
**Creatine kinase (U/L; normal range 50.0–310.0)**				0.80
Increased	1 (3.5)	0	1 (4.7)	
Normal	13 (44.8)	4 (50)	9 (42.9)	
Decreased	15 (51.7)	4 (50)	11 (52.4)	
**Lactate dehydrogenase (U/L; normal range 120.0–250.0)**				0.24
Increased	13 (44.8)	2 (25)	11 (52.4)	
Normal	16 (55.2)	6 (75)	10 (47.6)	
**Myoglobin (ng/mL; normal range 23.0–112.0)**				0.24
Increased	5 (17.3)	0	5 (23.8)	
Normal	19 (65.5)	7 (87.5)	12 (57.2)	
Decreased	5 (17.2)	1 (12.5)	4 (19)	
**Glucose (mmol/L; normal range 3.9–6.1)**				0.10
Increased	12 (41.4)	1 (12.5)	11 (52.3)	
Normal	16 (55.2)	7 (87.5)	9 (42.9)	
Decreased	1 (3.4)	0	1 (4.8)	
**BIOLOGICAL INDICATORS OF INFECTION, NO. (%)**				
**Procalcitonin (ng/mL; normal range 0–0.25)**				0.15
Increased	6 (20.7)	0	6 (28.6)	
Normal	23 (79.3)	8 (100)	15 (71.4)	
**Interleukin-6 (pg/mL; normal range 1.18–5.3)**				<0.05
Increased	16 (55.2)	1 (12.5)	15 (71.4)	
Normal	13 (44.8)	7 (87.5)	6 (28.6)	
**Erythrocyte sedimentation rate (mm/h; normal range 0.0–40.0)**				0.14
Increased	7 (24.1)	0	7 (33.3)	
Normal	22 (75.9)	8 (100)	14 (66.7)	
**Serum ferritin (ng/mL; normal range 21.8–274.1)**				0.26
Increased	16 (55.2)	4 (50)	12 (57.1)	
Normal	12 (41.4)	3 (37.5)	9 (42.9)	
Decreased	1 (3.4)	1 (12.5)	0	
**C-reactive protein (mg/L; normal range 0.0–10.0)**				1.00
Increased	23 (79.3)	6 (75)	17 (81)	
Normal	6 (20.7)	2 (25)	4 (19)	
Decreased	0	0	0	
**Co-infection, no. (%)**				0.14
Yes	7 (24.1)	0	7 (33.3)	
No	22 (75.9)	8 (100)	14 (66.7)	
**Co-infection type, no. (%)**				N/A
Bacteria	7 (24.1)	0	7 (33.3)	
Fungus	4 (13.8)	0	4 (19)	
Both	4 (13.8)	0	4 (19)	
**CT and chest x-ray imaging, no. (%)**				N/A
Unilateral pneumonia	5 (17.2)	1 (12.5)	4 (19)	
Bilateral pneumonia	6 (20.7)	0	6 (28.6)	
Multiple mottling and ground-glass opacity	19 (65.5)	6 (75)	13 (61.9)	

*COVID-19, coronavirus disease 2019; CT, computed tomography; N/A, not available*.

*P-values: chi-square or Fisher's exact tests to compare the moderate- and severe-disease groups*.

### Treatment Characteristics

Six of the 29 patients (6/29; 21%), were admitted to the ICU, all of whom had severe infections (Table 3). The majority of those patients (23/29; 80%) received oxygen therapy, antibiotics including cephalosporins, quinolones, and beta lactams, and 26 (26/29; 90%) received traditional Chinese medicines (TCMs), including Qing Fei Pai Du Tang, She Gan Ma Huang Tang, and Xiao Chai Hu Tang, which are recommended for pneumonia prevention and control by the China National Health Commission (15). Herbals, such as nutmeg, bitter almonds, licorice, Chen Pi (dried mandarin peel), ginger, honeysuckle, forsythia, pinellia, trichosanthes, and others were included in the prescriptions. The patients who received TCMs were discharged after 11.9 ± 7.0 days. Three patients without TCM treatment were discharged after 12.3 ± 3.2 days. The trend toward a shorter hospital stay with TCM is consistent with a previous report (16). When stratified by severity, patients with severe disease were more likely to need oxygen therapy, mechanical ventilation, intravascular immunoglobulin therapy, and an increased duration of antibiotic therapy (*P* < 0.05). Two patients with severe disease were treated with extracorporeal membrane oxygenation.

**Table 3:** Treatment of the COVID-19 patients.

	**All patients (*n* = 29)**	**Severity**		***P*-value**
		**Moderate (*n* = 8)**	**Severe (*n* = 21)**	
**Intensive care unit admission**, ***n*** **(%)**				0.14
Yes	6 (20.7)	0	6 (28.6)	
No	23 (79.3)	8 (100)	15 (71.4)	
**Oxygen therapy**, ***n*** **(%)**				<0.05
Yes	24 (82.8)	4 (50)	20 (95.2)	
No	5 (17.2)	4 (50)	1 (4.8)	
**Mechanical ventilation**, ***n*** **(%)**				<0.05
Non-invasive	19 (65.5)	4 (50)	15 (71.4)	
Invasive	5 (17.2)	0	5 (23.8)	
No	5 (17.2)	4 (50)	1 (4.8)	
**Antibiotics**, ***n*** **(%)**				0.65
Yes	23 (79.3)	7 (87.5)	16 (76.2)	
No	6 (20.7)	1 (12.5)	5 (23.8)	
Duration, median days (IQR)	7.0 (4.0–12.0)	3.0 (3.0–8.0)	8.5 (4.5–14.3)	<0.05
**Antifungal therapy**, ***n*** **(%)**				0.28
Yes	5 (17.2)	0	5 (23.8)	
No	24 (82.8)	8 (100)	16 (76.2)	
**Antiviral therapy, median days (IQR)**	10.0 (9.0–15.5)	11.5 (9.3–14.0)	10.0 (9.0–17.5)	0.94
**Glucocorticoids**, ***n*** **(%)**				0.24
Yes	13 (44.8)	2 (25)	11 (52.4)	
No	16 (55.2)	6 (75)	10 (47.6)	
Duration, median days (IQR)	5.0 (2.5–6.5)	3.5 (3.0–3.5)	5.0 (2.0–7.0)	0.49
**Intravascular immunoglobulin therapy**, ***n*** **(%)**				<0.05
Yes	12 (41.4)	0	12 (57.1)	
No	17 (58.6)	8 (100)	9 (42.9)	
**Extracorporeal membrane oxygenation**, ***n*** **(%)**				1.00
Yes	2 (6.9)	0	2 (9.5)	
No	27 (93.1)	8 (100)	19 (90.5)	
**Combined with traditional Chinese medicine**, ***n*** **(%)**				1.00
Yes	26 (89.7)	7 (87.5)	19 (90.5)	
No	3 (10.3)	1 (12.5)	2 (9.5)	

*COVID-19, coronavirus disease 2019; IQR, interquartile range; N/A, not available*.

*P-values: Mann–Whitney U-test, chi-square, or Fisher's exact test to compare the moderate and severe group*.

### Outcomes

Symptoms on admission and clinical outcomes of the COVID-19 patients are shown in Table 4. The three most common symptoms in patients with mild and severe disease on admission were fever, cough, and sputum production. Fever, cough, and fatigue were the three most common symptoms in patients with moderate disease. By March 10, 2020, 28 patients had been discharged and one had died. The patient who died was an 86-years-old woman who had been transferred to Henan Provincial People's Hospital. On admission, she had a lumbar spine tumor (benign or malignant unknown), hypertension, coagulopathy, multiple bone fractures caused by trauma 2 months previously, a 10-years history of untreated coronary heart disease, and sequelae of a cerebral infarction 4 years previously. When she was transferred, severe pneumonia had already developed. She was immediately admitted to the ICU, intubated for mechanical ventilation, and given antibiotics and antiviral treatment plus TCM. On the second day, 12 h of continuous renal replacement therapy was performed because of worsening renal damage. The patient soon developed a right atrial thrombus, deep vein thrombosis in both legs, severe respiratory failure, and heart failure. After sudden cardiac arrests on days 2 and 3 after admission, she was declared dead.

**Table 4:** Symptoms on admission and clinical outcomes of the COVID-19 patients (*n* = 29).

**Characteristic**		**Severity**		***P-*value**
		**Moderate**	**Severe**	
**Symptom on admission**, ***n*** **(%)**				N/A
Fever	26 (89.7)	7 (87.5)	19 (90.5)	
Cough	16 (55.2)	2 (25)	14 (66.7)	
Sputum production	10 (34.5)	2 (25)	8 (38.1)	
Shortness of breath	8 (27.6)	0	8 (38.1)	
Muscle ache	3 (10.3)	1 (12.5)	2 (9.5)	
Headache	3 (10.3)	2 (25)	1 (4.8)	
Sore throat	1 (3.4)	1 (12.5)	0	
Fatigue	8 (27.6)	4 (50)	4 (19)	
Diarrhea	1 (3.4)	0	1 (4.8)	
Nausea and vomiting	3 (10.3)	0	3 (14.3)	
Shiver	2 (6.9)	1 (12.5)	1 (4.8)	
**Clinical outcome**, ***n*** **(%)**				1.00
Discharged	28 (96.6)	8 (100)	20 (95.2)	
Died	1 (3.4)	0	1 (4.8)	

*COVID-19, coronavirus disease 2019; N/A, not available*.

*P-values: Fisher's exact test to compare the moderate and severe group*.

## Discussion

Henan province and Hubei province are not only border each other, but are also important transportation hubs and economic centers in the country. This geographical proximity and the frequent population exchanges provide an opportunity for the spreading of SARS-CoV-2. This descriptive study investigated the epidemiological and clinical characteristics of the first 29 patients with COVID-19 infection who were admitted to Henan Provincial People's Hospital, a hospital designated to admit confirmed patients from across Henan. The study revealed valuable information on the characteristics of the first severe COVID-19 infections diagnosed in Henan following the start of the outbreak in Hubei. It also includes outcomes in patients with treatment that included TCMs. Differences in some demographic and clinical characteristics of patients with mild, moderate, and severe disease were significant.

Previous coronavirus outbreaks, SARS-CoV in 2003 and MERS-CoV 2012, challenged the health care system of the country. SARS-COV resulted in 8,096 infections and 774 deaths, in 29 countries (17). SARS-CoV-2 is highly contagious in humans, with 823,626 infections and 40,598 deaths occurring in more than 200 countries by April 2020. The reported mortality of SARS-CoV has been reported as 5–10% and that of MERS-CoV as 35.7% (18, 19). Only one patient in this series (3.4%) died, which is lower than the 4.3–15% previously reported in Wuhan (20–22). Perhaps the severity of imported COVID-19 infections will be reduced compared with those diagnosed in Hubei province.

The patients in this series were between 20 and 90 years of age; the largest percentage (9/29; 31%) were middle-aged. Unlike a previous study in Wuhan that reported increased susceptibility in older men, this series included similar percentages of men and women (21). The largest proportion of patients (11/29; 38%) were agricultural workers, the others had diverse occupations. Four patients, two doctors and two nurses, worked in local hospitals and provided care for patients with confirmed COVID-19 infections, which supports the interpersonal transmission of SARS-CoV-2. The median hospital stay was 12 (IQR, 10–16; range, 3–35) days. The only death was of an 86-years-old woman on the third day after admission, and it resulted from progressive respiratory failure and two cardiac arrests. Twenty-eight patients had recent histories of contact with confirmed or probable COVID-19 infection. Four were health care personnel, and except for a 61-years-old woman, the remaining 24 had returned from travel to Hubei province. The patient histories are in line with evidence of possible asymptomatic patients described by Bai et al. (23), and considered to be a risk factor for subsequent outbreaks (24, 25). The transmission of SARS-CoV-2 by asymptomatic individuals is not fully understood and requires investigation.

The most prevalent COVID-19 symptom in this case series was an initial fever that was often accompanied by cough and sputum production. Some older patients presented with shortness of breath or fatigue. Although 26 of the 29 patients had initially experienced fevers, the atypical presenting symptoms in the other three patients cannot be ignored. They were admitted with cough and sputum production, diarrhea, nausea and vomiting, and a sore throat that may not be the usual signs of COVID-19 infection but need consideration.

TCM was found to be effective for treating SARS in 2003 (26). Many ingredients in the mixtures used to treat the COVID-19 infections in this series are routinely used to treat colds, fever, or cough (27). The patients in this series were treated with decoctions, Chinese patent medicines, or both, depending on syndrome differentiation, and had a shorter hospital stay than the other patients. Twenty-three patients were given oxygen therapy on admission in contrast to a previous report that only 1.6% patients needed ICU admission and oxygen therapy (28). Twenty-one patients in this investigation were admitted or transferred to ICU because they required continuous high-flow oxygen therapy, CRRT or ECMO. Antiviral treatment included inhaled interferon alpha (12 million IU/day) for all 29 patients. Oral arbidol (umifenovir) 0.6 g/day), intravenous ribavirin (1.0 g/day), oral lopinavir and ritonavir (800/200 mg/day), or oral chloroquine phosphate (1.0 g/day) were included in the treatment of severely ill patients. Many patients received antibiotic prophylaxis. Glucocorticoids, including methylprednisolone sodium succinate, methylprednisolone, and dexamethasone were given to patients with ARDS or critical respiratory failure for relatively short times to minimize adverse reactions. Some patients received intravenous immunoglobulin therapy because of immune deficiencies.

### Study Limitations

The study limitations include the inclusion of cases treated in only one of the three hospitals designated to treat COVID-19 patients in Henan. It is possible that this group of patients does not completely represent the characteristics of infections diagnosed across the province. Also, the inclusion of patients who were transferred from other hospitals may have resulted in bias because of the collection of inaccurate data in the early stages of infection. Secondly, the viral load, which is likely to influence the severity of infection was not included in the analysis. Thirdly, although the patients were stratified by the severity of their clinical and epidemiological characteristics, it was difficult to analyze the association of differences between variables and severity because the load of infection was not determined. Finally, the small sample size makes it difficult to assure an accurate mortality rate or identify potential exposures and risk factors that can trigger the infection. Subsequent studies with larger sample sizes will answer these questions. The study was not an extended investigation of COVID-19 cases in Henan, but its value is in providing early patient and epidemiological data that add to what is known of this emerging viral disease and will be of use in the ongoing effort to control this pandemic.

## Conclusion

The clinical and epidemiological features of these 29 COVID-19 patients in Henan show that the virus tended to infect middle-aged and older people with underlying diseases. About 21% of the patients were admitted to the ICU, the median hospital stay was 12 days, and the mortality rate was 3.4%. Some differences of the clinical and epidemiological features between patients with moderate and severe disease were significant.

## Data Availability Statement

The raw data supporting the conclusions of this article will be made available by the authors, without undue reservation.

## Ethics Statement

The studies involving human participants were reviewed and approved by the Ethics Commission of the Henan Provincial People's Hospital. Written informed consent for participation was not required for this study in accordance with the national legislation and the institutional requirements.

## Author Contributions

Q-JW and JZ conceived and conceptualized the study. H-YG and Z-YF wrote the initial draft of the article with important feedback from Q-JW and JZ that affected the subsequent revisions. All authors were involved in the editing the article, and agree on the final version.

## Conflict of Interest

The authors declare that the research was conducted in the absence of any commercial or financial relationships that could be construed as a potential conflict of interest.
